
JOURNAL INFORMATION
==============================
NLM Title Abbreviation: Buffalo Med J
NLM Journal ID: 0027534

Buffalo Medical Journal

ISSN: 1040-3817
Publisher: Biomedical Journal Digitization Project

ARTICLE INFORMATION
==============================
PMCID: PMC8731670
PMID: 36886158
Article version: 1
Subjects: Item

Item

Print publication date: 1903 Jul
Volume: 42
Volume Series: New Ser
Issue: 12
First page: 948
Last page: 948
License: This work is free of known copyright restrictions. For more information, please see PMC Back Issue Digitization.
License URL: https://creativecommons.org/publicdomain/mark/1.0/

==============================

Volume XXI.—Supplement. New York: William Wood & Co. 1903. (Price, cloth, $5.00.)

Surgical Diseases of the Abdomen. With special reference to diagnosis. By Richard Douglas, M. D. Formerly Professor of Gynecology and Abdominal Surgery, Medical Department, Vanderbilt University, Nashville. Octavo, pp. 883. Illustrated by 20 full-page plates. Philadelphia: P. Blakiston's Son & Co. 1903. (Price, cloth, gilt top, $7.00.)

### LITERARY NOTES.

The Batavia Woman's Hospital Association, the corporate body under the auspices of which the Batavia hospital is managed, has issued its first report which embraces the period from May, 1900, to July 15, 1902. The report shows in detail the splendid work that has been done in establishing one of the finest small hospitals in Western New York, which is a credit to every person who has contributed time, material, or money toward its completion and maintenance.

Battle & Company, of Saint Louis, have issued chart No. 2, illustrating fractures of long bones. This is the last of the series and presents Colles's fracture—fracture of the lower end of the radius—in a graphic manner. The contraction of the supinator longus acts to tilt the fracture against and thus displace the lower end of the ulna. This could have been made a more prominent feature of the drawing; nevertheless, it is an instructive delineation of this classic injury.

### ITEM.

The Maltine Company has published a very convenient and valuable chart of the principal poisons and their antidotes, which will be promptly sent to physicians and to hospitals, dispensaries, training schools for nurses and kindred institutions on application. Address, The Maltine Company, 8th Avenue and 13th Street, Brooklyn, N. Y.

FOR SALE—A beautiful, well-equipped, paying Surgical Sanitarium and Hospital, located in Western New York, in a rich agricultural and natural gas belt. Brick and stone structure, with all modern improvements. All kinds of fruit, good barn, and an acre of land. A great opportunity. Terms low.

Address Buffalo Medical Journal,
284 Franklin Street, Buffalo, N. Y.
